# Transmission through seeds: The unknown life of plant viruses

**DOI:** 10.1371/journal.ppat.1010707

**Published:** 2022-08-11

**Authors:** Israel Pagán

**Affiliations:** Centro de Biotecnología y Genómica de Plantas UPM-INIA/CSIC and Departamento de Biotecnología-Biología Vegetal, E.T.S. Ingeniería Agronómica, Alimentaria y de Biosistemas, Universidad Politécnica de Madrid, Madrid, Spain; Shanghai Center for Plant Stress Biology, CHINA

## Introduction

Transmission efficiency is a key trait for every pathogen as it ultimately determines fitness. Plant viruses are not exceptions and evolved various ways to achieve transmission. The best characterized is plant-to-plant horizontal transmission by direct contact, mechanical means (lawn and farm equipment, grazing animals, etc.) or by vectors (arthropods, nematodes, fungi, oomycetes, etc.). While very important for the epidemiology of most plant viruses, horizontal transmission is far from being the only way for virus dispersal. Parent-to-offspring vertical transmission through seeds has been known for a century [1], and for some plant viruses such as persistent (also known as cryptic), viruses is the only way to infect new hosts [2]. However, many aspects of the virus journey to reach the reproductive organs and its life within seeds remain poorly understood. This knowledge gap can be perhaps attributed to a combination of factors: Due to the low virus titer in the seed, detection is often difficult. Also, studies on seed transmission require long-term experiments, which is frequently a limiting factor for researchers. Consequently, until recently, it was considered as a transmission mode of lesser relevance. For instance, 35 years ago, only 100 plant viruses were described as seed-transmitted, normally at very low rates [3]. This vision is currently changing. Methods for virus detection in seeds and seedlings (virus detection in seeds does not necessarily entail transmission to the progeny, as virus could be present in the testa/endosperm but not in the embryo) have been progressively improved in specificity and sensitivity [4]. In parallel, the number of viruses described as seed-transmitted increased 6-fold, with some reaching 100% of infected seeds [2,5]. Moreover, it has been shown that vertically transmitted persistent viruses are predominant in wild plants, and they are also common in crops [2]. By answering 5 basic questions about plant virus seed transmission, here I aim to provide arguments supporting why we should care about this mode of transmission and to summarize current knowledge and future avenues of research on this subject.

## Does seed transmission contribute to virus epidemics?

**Fig 1:**
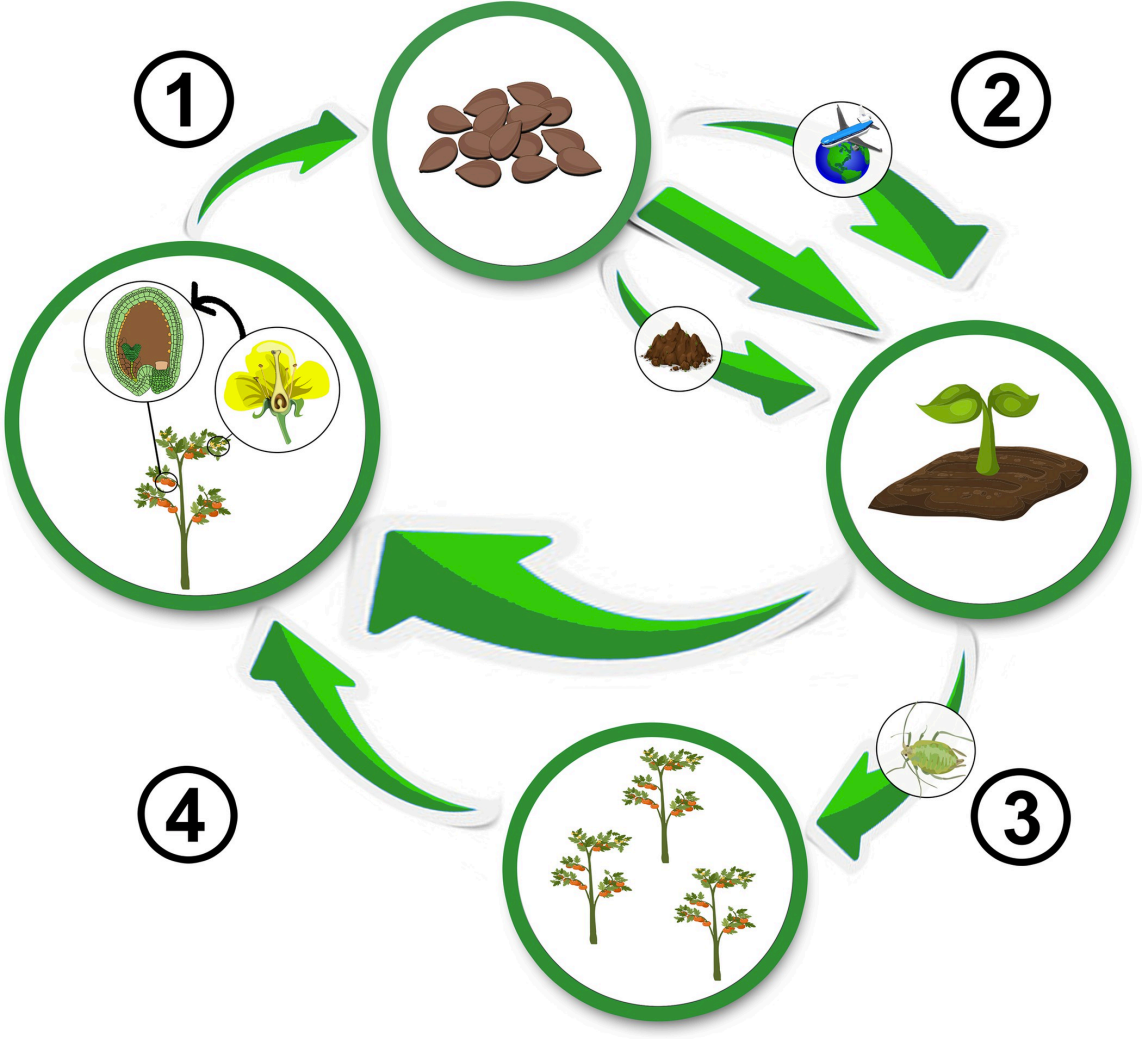
Virus seed transmission cycle. (1) Plant viruses reach seeds either by direct invasion of the embryo from the parental plant via the suspensor, and/or indirectly by infecting pollen grains or ovules, which lead to an infected embryo after fertilization. (2) Infected seeds may (i) directly germinate; (ii) remain for long periods of time in the soil; or (iii) be dispersed to long distances. In any of these 3 scenarios, infected seed will produce infected seedlings after germination. (3) Infected seedlings (and adult plants) from infected seeds will be sources of primary inoculum allowing virus dispersion through vectors. The picture represents aphids, which transmit 30% of plant viruses. (4) The cycle can be closed in 2 ways: (i) Viruses from infected seedlings/adult plants may again reach seeds leading to a second round of seed transmission, allowing virus persistence in the absence of vectors. (ii) Plants infected by vectors can produce infected seeds. Constructed using BioRender.

Seed infection provides the virus with means to persist for long periods of time when hosts and/or vectors are not available, as many seed-transmitted viruses can survive within the seed as long as it remains viable [6]. Seed transmission also allows for long-distance dissemination of plant viruses, and evidence exist that bird dispersion and human trade of infected seeds resulted in cross-continental virus jumps (for instance, [7]). But perhaps the most important epidemiological effect of seed transmission is that it represents an important source of primary inoculum for many viruses with vertical transmission, which are disseminated afterwards via vectors causing devastating epidemics (**Fig 1**). This is particularly important as many vectors, particularly aphids, transmit viruses in a nonpersistent manner (virus acquisition and further inoculation in susceptible plants occur within a few seconds/minutes), which means that insecticides are not effective at suppressing virus spread [8]. In the current context of accelerating climate change, virus seed transmission may confer crucial benefits for plant viruses: It allows virus survival in unfavorable conditions, such as prolonged drought periods, and facilitates virus geographic range expansion when climatic conditions become favorable in distant regions that viruliferous vectors would not reach [9]. Indeed, it has been shown that elevated temperature and light intensity, both associated with global warming, increase the survival of infected seeds and seed transmission rate [10,11]. Thus, seed transmission is (and likely will be) of great importance in plant virus epidemics and of major concern for food safety as demonstrated by the numerous national and international regulations on seed phytosanitary measures [12].

## How does the mode of transmission affect virus evolution?

Due to its central role in determining virus fitness, a considerable body of theory is aimed at predicting the conditions in which pathogen transmission will be optimized. Applied to plant viruses, most of these works agree in that maximum seed transmission rates require optimal plant reproduction and, therefore, lower virulence (i.e., effect of infection on seed number) [13]. The scarce experimental analyses, in which serial virus passages through strict vertical transmission resulted in higher rates of infected seeds and reductions of virus accumulation and virulence, support this prediction [14,15]. Interestingly, optimizing seed transmission also benefits the host plant as the virus may evolve toward commensalism (higher plant fitness). Accordingly, plant adaptation to this mode of transmission has been reported [15]. Hence, although limited, evidence indicates that seed transmission may play a central role in both plant and virus evolution.

## Can we reduce the impact of virus seed transmission?

Current strategies to minimize the impact of seed-transmitted viruses mostly involve routine seed health testing and, to a lesser extent, heat treatment of infected seeds [4]. However, these methods are of limited use as realistic thresholds of virus infection have been investigated only for a few plant–virus interactions [16]. Consequently, in most cases, the acceptable level of virus seed infection remains debatable, and it may be well above the minimum primary inoculum necessary to start an epidemic. For instance, in Europe, the acceptable threshold limit of lettuce seeds infected by *Lettuce mosaic virus* was 0.1%, until it was demonstrated that a percentage of infected seeds as low as 0.003% was enough to start an epidemic [4]. A similar limitation applies for methods based on heat treatment of infected seeds, as these may reduce virus incidence but rarely result in complete eradication [4]. In addition, these methods compromise seed viability [17]. Strategies to regenerate virus-free plants in vitro from meristems are increasingly available, but they still cover a limited number of cultivated plants [18]. The deployment of plant genetic resistance, which is a broadly used strategy to control virus diseases, could be a good alternative to current control methods, but it is only available for a reduced number of plant viruses and its durability is limited [19]. Perhaps specific genetic resistance to seed transmission, more broadly found across plant species than immunity to infection [5], could contribute to reduce the impact of this mode of transmission in virus epidemics: It would allow the production of virus-free certified seeds and reduce the sources of primary inoculum. However, this strategy would require extensive knowledge on the mechanisms of seed transmission, which is currently lacking.

## How are viruses transmitted through seeds?

Seed-transmitted viruses have 2 nonmutually exclusive mechanisms to reach the seed. First, direct invasion of the embryonic tissue, and second, infection of the ovules and/or the pollen (**Fig 1**). To understand these routes of transmission, it should be noted that, unlike animal viruses where receptor-mediated mechanisms facilitate exploiting the extracellular environment for within-host dissemination, plant viruses are restricted to the intracellular compartment (symplast). Their cell-to-cell movement occurs through symplastic channels (plasmodesmata), a process mediated by viral *movement proteins* [20]. Hence, embryo or gamete invasion requires a symplastic connection with the parental plant [18]. In the case of direct embryo invasion, infection occurs after fertilization through the suspensor, which provides nutritional and positional support for the embryo, and temporarily maintains this symplastic connection until its programmed cell death [21]. Seed transmission through gamete infection requires invasion during gametogenesis prior to fertilization, which also occurs via plasmodesmata. In autogamous species (i.e., species in which ovules are predominantly fertilized by pollen from the same flower), the virus must be present in the parental plant. However, in plants with sexual dimorphism, pollen infection allows seed transmission in uninfected females, although less efficiently than seed infection from the parental plant [6]. It should be mentioned that a third route of seed transmission via host cell meiosis has been proposed for persistent viruses, which apparently do not move systemically [22]. Whatever the mechanism, it is well established that it is controlled by both the host plant and the virus [23].

## What is known about the molecular basis of seed transmission?

According to current knowledge on the mechanisms of seed transmission, it has been predicted that plant and virus genetic determinants would control the following: (i) virus multiplication and movement; (ii) virus ability to invade gametic tissues; and (iii) gamete and embryo survival in the presence of the virus [20].

There is remarkably little information on the function of plant genes involved in virus seed transmission. In soybean, *Soybean mosaic virus* seed transmission is controlled by host genes homolog of Arabidopsis *DCL3* and *RDR6*, which are involved in the RNA silencing plant defense response [24]. Also, in barley and pea, it has been shown that seed transmission of *Barley stripe mosaic virus* (BSMV) and *Pea seed-borne mosaic virus* (PSbMV), respectively, is controlled by a few unidentified loci of quantitative effect [21]. In Arabidopsis, genome-wide association studies have identified genes related to plant response to stresses, embryogenesis, and cell wall metabolism (involved in virus within-host movement) as associated with *Cucumber mosaic virus* (CMV) seed transmission [25]. These in silico analyses need to be experimentally validated, but functions of the identified genes agree with predictions above.

Virus genetic determinants involved in seed transmission have been analyzed in a handful of species and are also associated with virus multiplication, movement, and invasion of plant reproductive organs [23]. Determinants of seed transmission identified in the *Pea early browning virus* and CMV genomes are in RNAs 1 and 2, which encode proteins involved in virus replication, and in the 12-kDa gene, which is an RNA silencing suppressor [26,27]. Similarly, for PSbMV and BSMV, the HC-Pro and the γb proteins that are suppressors of the RNA silencing plant defense response, as well as mutations in other regions that are important for virus multiplication and movement, affect virus seed transmission rate [28–30].

## Concluding remarks

In sum, plant viruses are not restricted to host vegetative organs and vectors, but also invade reproductive organs and seeds. Exploring this largely unknown aspect of the life of plant viruses will yield key information to understand their epidemiology and evolution.

## References

[1] Doolittle SP. The mosaic disease of cucurbits. US Dep Agric Bull. 1920;879:1–69.

[2] Roossinck MJ. Plants, viruses and the environment: Ecology and mutualism. Virology. 2015;479–480:271–277. doi: 10.1016/j.virol.2015.03.041 25858141

[3] Stace-Smith R, Hamilton RI. Inoculum threshold of seedborne pathogens. Phytopathology. 1988;78:875–880.

[4] Aveling TAS. Global standards in seed health testing. In: Gullino ML, Munkvold G, editors. Global Perspectives on the Health of Seeds and Plant Propagation Material. Dordrecht: Springer; 2014. p. 17–28.

[5] Pagán I. Movement between plants: Vertical transmission. In: Palukaitis P, García-Arenal F, editors. Cucumber Mosaic Virus. Washington: APS Press; 2019. p. 185–198.

[6] Sastry KS. Seed-borne plant virus diseases. New Delhi: Springer; 2013.

[7] Dwyer GI, Gibbs MJ, Gibbs AJ, Jones RAC. *Wheat streak mosaic virus* in Australia: relationship to isolates from the Pacific Northwest of the USA and its dispersion via seed transmission. Plant Dis. 2007;91:164–170. doi: 10.1094/PDIS-91-2-0164 30780999

[8] Perring TM, Gruenhagen NM, Farrar CA. Management of plant viral diseases through chemical control of insect vectors. Annu Rev Entomol. 1999;44:457–481. doi: 10.1146/annurev.ento.44.1.457 15012379

[9] Jones RAC. Future scenarios for plant virus pathogens as climate change progresses. Adv Virus Res. 2016;95:87–147. doi: 10.1016/bs.aivir.2016.02.004 27112281

[10] Bueso E, Serrano R, Pallás V, Sánchez-Navarro JA. Seed tolerance to deterioration in arabidopsis is affected by virus infection. Plant Physiol Biochem. 2017;116:1–8. doi: 10.1016/j.plaphy.2017.04.020 28477474

[11] Montes N, Pagán I. Light intensity modulates the efficiency of virus seed transmission through modifications of plant tolerance. Plan Theory. 2019;8:304. doi: 10.3390/plants8090304 31461899 PMC6783938

[12] Food and Agriculture Organization of the United Nations. International Standards for Phytosanitary Measures 38. International Movement of Seeds. Rome: FAO; 2021.

[13] Lipsitch M, Siller S, Nowak MA. The evolution of virulence in pathogens with vertical and horizontal transmission. Evolution. 1996;50:1729–1741. doi: 10.1111/j.1558-5646.1996.tb03560.x 28565576

[14] Stewart AD, Logsdon JM, Kelley SE. An empirical study of the evolution of virulence under both horizontal and vertical transmission. Evolution. 2005;59:730–739. 15926685

[15] Pagán I, Montes N, Milgroom MG, García-Arenal F. Vertical transmission selects for reduced virulence in a plant virus and for increased resistance in the host. PLoS Pathog. 2014;10:e1004293. doi: 10.1371/journal.ppat.1004293 25077948 PMC4117603

[16] Coutts BA, Prince RT, Jones RA. Quantifying effects of seedborne inoculum on virus spread, yield losses, and seed infection in the *Pea seed-borne mosaic virus*-field pea pathosystem. Phytopathology. 2009;99:1156–1167. doi: 10.1094/PHYTO-99-10-1156 19740029

[17] Paylan IC, Erkan S, Cetinkaya N, Ergu M, Pazarlar S. Effects of different treatments on the inactivation of various seedborne viruses in some vegetables. Ozone Sci Eng. 2014;36:422.

[18] Bradamante G, Mittelsten Scheid, O, Incarbone M. Under siege: virus control in plant meristems and progeny, Plant Cell. 2021;33:2523–2537. doi: 10.1093/plcell/koab140 34015140 PMC8408453

[19] García-Arenal F, McDonald BA. An analysis of the durability of resistance to plant viruses. Phytopathology. 2003;93:941–952. doi: 10.1094/PHYTO.2003.93.8.941 18943860

[20] Hull R. Plant Virology. 5th ed. London: Academic Press; 2014.

[21] Maule AJ, Wang D. Seed transmission of plant viruses: a lesson in biological complexity. Trends Microbiol. 1996;4:153–158. doi: 10.1016/0966-842x(96)10016-0 8728609

[22] Boccardo G, Lisa V, Luisoni E, Milne RG. Cryptic plant viruses. Adv Virus Res. 1987;32:171–214. doi: 10.1016/s0065-3527(08)60477-7 3303860

[23] Cobos A, Montes N, López-Herranz M, Gil-Valle M, Pagán I. Within-host multiplication and speed of colonization as infection traits associated with plant virus vertical transmission. J Virol. 2019;93:e01078–19. doi: 10.1128/JVI.01078-19 31511374 PMC6854480

[24] Domier LL, Hobbs HA, McCoppin NK, Bowen CR, Steinlage TA, Chang S, et al. Multiple loci condition seed transmission of *Soybean mosaic virus* (SMV) and SMV-induced seed coat mottling in soybean. Phytopathology. 2011;101:750–756. doi: 10.1094/PHYTO-09-10-0239 21561316

[25] Montes N, Cobos A, Gil-Valle M, Caro E, Pagán I. *Arabidopsis thaliana* genes associated with *Cucumber mosaic virus* virulence and their link to virus seed transmission. Microorganisms. 2021;9:692. doi: 10.3390/microorganisms9040692 33801693 PMC8067046

[26] Hampton RO, Francki RIB. RNA-1 dependent seed transmissibility of cucumber mosaic virus in *Phaseolus vulgaris*. Phytopathology. 1992;82:127–130.

[27] Wang D, MacFarlane SA, Maule AJ. Viral determinants of pea early browning seed transmission in pea. Virology. 1997;234:112–117. doi: 10.1006/viro.1997.8637 9234951

[28] Edwards MC. Mapping of the seed transmission determinants of barley stripe mosaic virus. Mol Plant Microbe Interact. 1995;8:906–915. doi: 10.1094/mpmi-8-0906 8664501

[29] Johansen IE, Dougherty WG, Keller KE, Wang D, Hampton RO. Multiple viral determinants affect seed transmission of pea seedborne mosaic virus in *Pisum sativum*. J Gen Virol. 1996;77:3149–3154. doi: 10.1099/0022-1317-77-12-3149 9000110

[30] Roberts IM, Wang D, Thomas CL, Maule AJ. *Pea seed-borne mosaic virus* seed transmission exploits novel symplastic pathways to infect the pea embryo and is, in part, dependent upon chance. Protoplasma. 2003;222:31–43. doi: 10.1007/s00709-003-0015-5 14513309

